# *In vivo* safety profile of a CSPG4-directed IgE antibody in an immunocompetent rat model

**DOI:** 10.1080/19420862.2019.1685349

**Published:** 2019-11-26

**Authors:** Iwan P. Williams, Silvia Crescioli, Heng Sheng Sow, Heather J. Bax, Carl Hobbs, Kristina M. Ilieva, Elise French, Giulia Pellizzari, Vivienne Cox, Debra H. Josephs, James F. Spicer, Sophia N. Karagiannis, Silvia Mele

**Affiliations:** a St John`s Institute of Dermatology, School of Basic and Medical Biosciences, King`s College London, London, UK; b IGEM Therapeutics Ltd, London BioScience Innovation Centre, London, UK; c Wolfson Centre for Age-Related Diseases, King’s College London, London, UK; d Breast Cancer Now Research Unit, School of Cancer & Pharmaceutical Sciences, King’s College London, Guy’s Cancer Centre, London, UK; e School of Cancer & Pharmaceutical Sciences, King’s College London, Bermondsey Wing, Guy’s Hospital, Bermondsey Wing, London, UK; f Department of Medical Oncology, Guy’s and St Thomas’ NHS Foundation Trust, Guy`s Hospital, London, UK; g Guy’s and St Thomas’ NHS Foundation Trust, Department of Oncology, Guy`s Hospital, Bermondsey Wing, London, UK

**Keywords:** IgE, rat model, immunotherapy, allergooncology, CSPG4, antibody, species cross-reactivity

## Abstract

IgE monoclonal antibodies hold great potential for cancer therapy. Preclinical *in vivo* systems, particularly those in which the antibody recognizes the host species target antigen and binds to cognate Fc receptors, are often the closest approximation to human exposure and represent a key challenge for evaluating the safety of antibody-based therapies. We sought to develop an immunocompetent rat system to assess the safety of a rodent anti-tumor IgE, as a surrogate for the human therapeutic candidate. We generated a rat IgE against the human tumor-associated antigen chondroitin sulfate proteoglycan 4 (CSPG4) and cross-reactive for the rat antigen. We analyzed CSPG4 distribution in normal rat and human tissues and investigated the *in vivo* safety of the antibody by monitoring clinical signs and molecular biomarkers after systemic administration to immunocompetent rats. Human and rat CSPG4 expression in normal tissues were comparable. Animals receiving antibody exhibited transient mild to moderate adverse events accompanied by mild elevation of serum tryptase, but not of angiotensin II or cytokines implicated in allergic reactions or cytokine storm. In the long term, repeated antibody administration was well tolerated, with no changes in animal body weight, liver and kidney functions or blood cell counts. This model provides preclinical support for the safety profiling of IgE therapeutic antibodies. Due to the comparable antigen tissue distribution in human and rat, this model may also comprise an appropriate tool for proof-of-concept safety evaluations of different treatment approaches targeting CSPG4.

## Introduction

Using recombinant monoclonal antibodies (mAb) of the IgE class for immunotherapy of cancer is an innovative approach that has shown promising results *in vitro* and *in vivo*.^1–3^ MAbs for the treatment of cancer are typically designed as IgGs, but use of such molecules has drawbacks, including: 1) relatively low affinity for cognate Fcγ receptors; 2) short half-life in tissues; and 3) the expression of inhibitory Fcγ receptors (FcγRIIb) on immune cells in the tumor microenvironment (TME), which may limit the effector functions and potency of IgGs to promote immune surveillance against solid tumors.^4,5^ In contrast, IgE displays up to 10,000-fold higher affinities for and slow dissociation from cognate Fcϵ receptors on IgE effector cells often found in the TME, lacks inhibitory FcRs, is retained for longer than IgG in tissues, and can exert immune surveillance in Th2-biased environments such as the TME.^2,5–8^ These attributes of IgE, actively investigated in the field of AllergoOncology, may be harnessed for cancer immunotherapy.^1,2,9^ Several studies in disparate animal models have demonstrated the anticancer efficacy of IgE mAbs directed to tumor antigens, including immunocompetent human FcϵRIα transgenic mice treated with chimeric or humanized IgE antibodies,^10–12^ syngeneic murine and rat carcinoma models,^13–15^ and immunocompromised SCID mouse models bearing human carcinoma xenografts.^16–18^ When compared to their IgG counterparts, IgE mAbs showed superior anti-tumor efficacy,^10,16–19^ which could be at least partly due to the IgE-mediated reprogramming of monocytes and macrophages within the tumor microenvironment.^18–22^ A first-in-human Phase 1 study (NCT02546921) of MOv18 IgE, a chimeric IgE antibody against folate receptor-alpha (FRα), has been initiated at King’s College London sponsored by Cancer Research UK. However, the pivotal role of IgE antibodies in the allergic response and systemic hypersensitivity reactions represents the main concern in the use of IgE mAbs for passive immunotherapy.

Due to the multimodal activity of therapeutic mAbs, it is challenging to find one animal model that can recapitulate the complexity of the tumor microenvironment, the human immune response and the multifaceted mechanisms of action of mAbs whilst concurrently allowing a comprehensive analysis of safety. Preclinical models for the study of therapeutic antibody safety should ideally offer: 1) an intact immune system that can mimic the expression and binding of human Fc receptors in the relevant immune cell populations; 2) a relevant tissue distribution of the target antigen; and 3) cross-reactivity of the antibody with orthologues of its respective human target.

Murine models are not considered representative of human IgE biology, primarily because of the substantial inter-species differences in the structure and expression pattern of the high-affinity IgE receptor.^23^ Conversely, rat FcϵRI shares distinct features with human FcϵRI that makes it a more suitable rodent model for the study of IgE-mediated cancer immunotherapy.^1,15^ In particular, unlike mouse, both rat and human FcϵRI can be expressed either as a trimeric (αγ2) or a tetrameric form (αβγ2), and expression is not restricted to basophils and mast cells but it can be found in other immune cells such as eosinophils, dendritic cells and macrophages.^24^ Previous animal models used to test IgE passive immunotherapy did not report antibody cross-reactivity between the target human antigen and the orthologue of the host species, with the exception of a pilot study where a single infusion of 0.08 mg/kg anti-HER2/*neu* IgE was injected into a cynomolgus monkey.^12^ The clinical translation of IgE immunotherapy in solid cancers is now moving forward, and further studies are needed to elucidate its safety profile and applicability across different tumor types and target antigens.

Chondroitin-sulfate proteoglycan 4 (CSPG4), also known as neuronal-glial antigen 2 (NG2), is considered a promising candidate target for cancer immunotherapy because of its diffuse and high level expression in a broad range of tumor types, such as melanoma, glioblastoma and subsets of breast carcinomas.^25^

Here, we designed a rat model to study the safety of a monoclonal IgE antibody directed against CSPG4. The distribution of human CSPG4 and its rat orthologue were evaluated in normal human and rat tissues. Taking advantage of the ability of a murine anti-CSPG4 antibody (clone 225.28) to cross-react with human CSPG4 and its rat orthologue, we generated a surrogate rat IgE mAb, α-CSPG4 rIgE, and looked for *in vivo* immediate and long-term adverse effects in immunocompetent rats through analysis of clinical signs and molecular biomarkers.

## Results

### CSPG4 distribution in rat and human normal tissues

**Figure 1.**
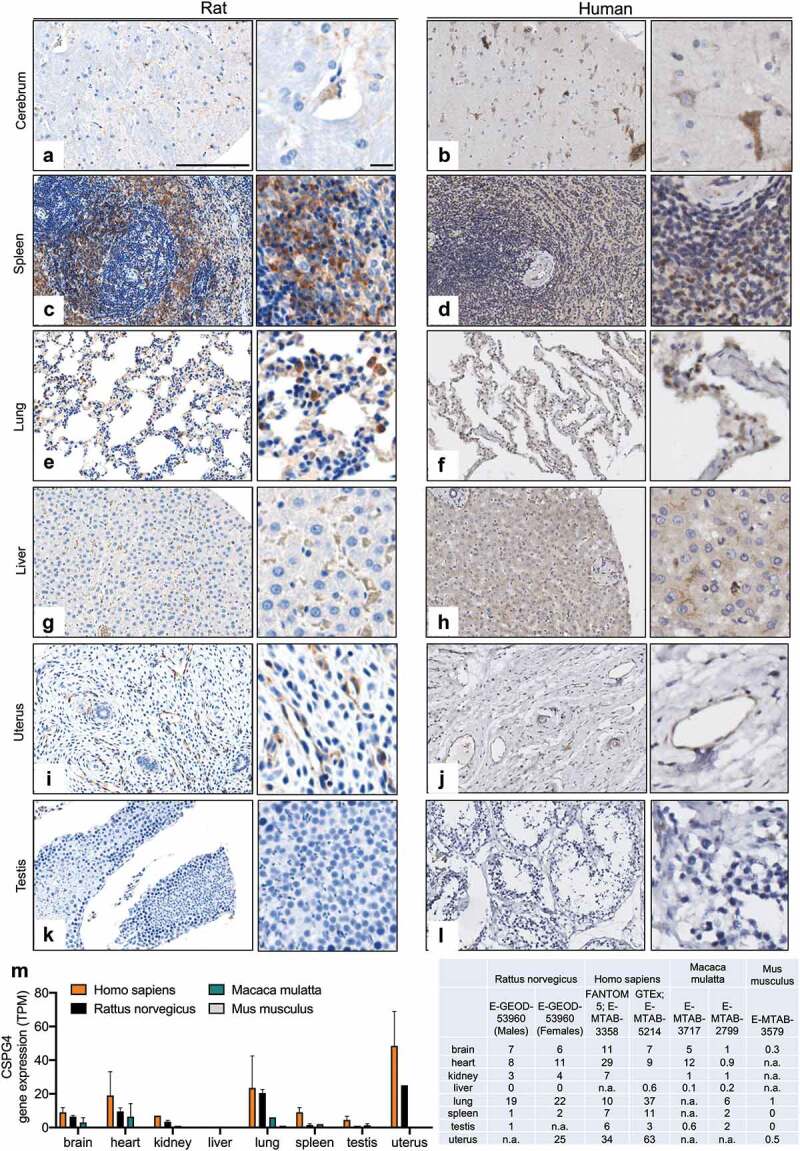
CSPG4 expression in normal human and rat tissues. (**A-L**) CSPG4 expression in rat (left) and human (right) tissues was investigated by immunohistochemistry of tissue microarrays using commercial anti-CSPG4 antibodies and developed using DAB chromogen. Hematoxylin was used to counterstain. Scale bars represent 200 μm (lower magnification) and 20 μm (higher magnification). **M**, Histogram (left) summarizing CSPG4 gene expression (Transcripts Per Million, TPM) in the specified tissues of four different species based on the transcriptomic dataset (EMBL-EBI Expression Atlas, https://www.ebi.ac.uk/gxa/). Dataset and respective mRNA expressions are listed in the table (right); n.a.: data not available.

In order to test the relevance of a rat model in the context of the safety of a CSPG4-targeted antibody, we compared the distribution of CSPG4 across a range of human and rat normal tissues. Similar patterns of CSPG4 expression were observed between the two species (Figure 1A–L). In agreement with studies that have reported the expression of CSPG4 in oligodendrocyte progenitor cells of the central nervous system (CNS),^26,27^ we detected CSPG4-positive cells in rat and human cerebrum (Figure 1A,B). In lung and liver tissues, we observed scattered cells with a moderate expression of CSPG4, whereas low expression was detected in pneumocytes (Figure 1E, F) and hepatocytes (Figure 1G, H). In line with previous studies that identified CSPG4 as a marker of angiogenetic vasculature,^28–30^ we observed CSPG4 expression along blood vessels in rat and human uterus tissues (Figure 1I, J). Human bone marrow, thyroid gland and adenohypophysis showed moderate CSPG4 expression, whereas no expression was detected in human peripheral nerve, cerebellum and esophagus tissues (Supplemental Figure 2, data for the respective rat tissues are not available). Moreover, when comparing CSPG4 gene expression in normal tissues of four different species through interrogation of transcriptomic datasets (EMBL-EBI Expression Atlas), human and rat showed similar expression profiles (Figure 1M).

**Figure 2.**
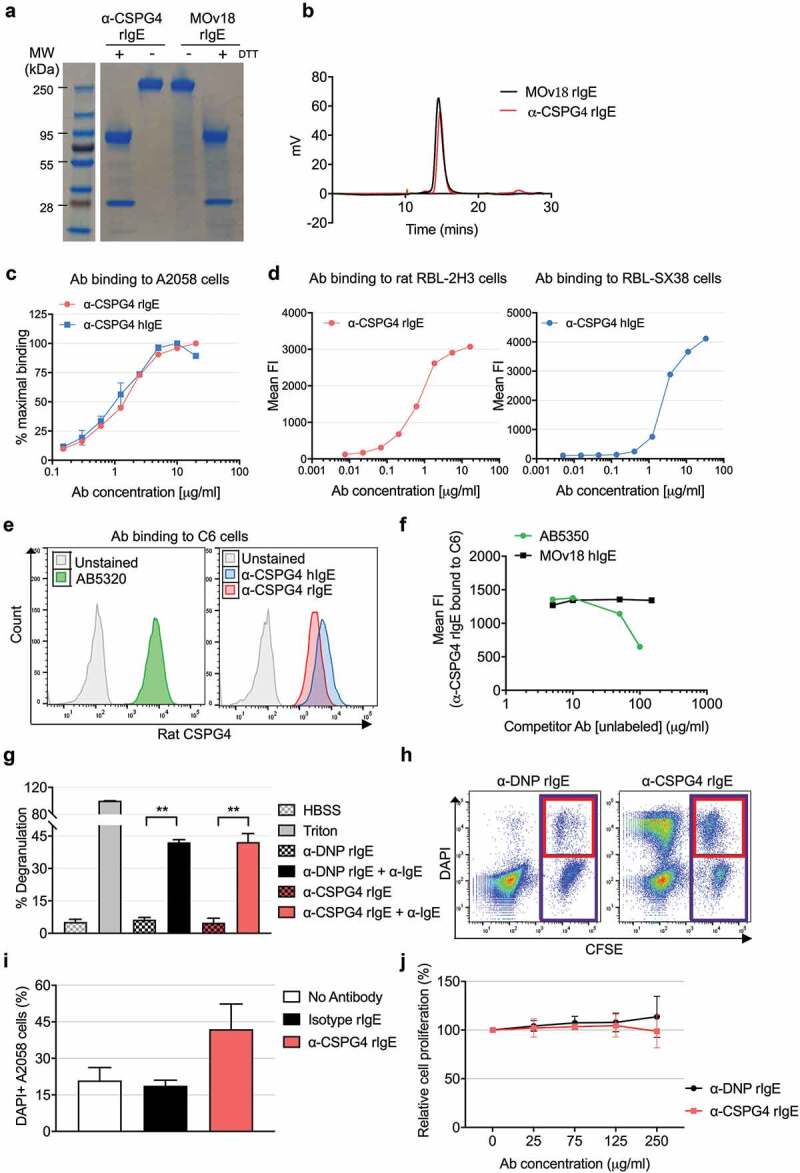
In vitro characterization of α-CSPG4 rIgE antibody. A, SDS-PAGE of reduced (DTT+) and non-reduced (DTT-) α-CSPG4 rIgE and MOv18 rIgE. B, HPLC-SEC profile of α-CSPG4 rIgE and MOv18 rIgE. C, Dose-dependent binding of α-CSPG4 rIgE (left) and α-CSPG4 hIgE (right) to CSPG4-expressing human A2058 cells detected by flow cytometry and expressed as % of maximal binding (maximal Mean Fluorescence Intensity). D, Dose-dependent binding of α-CSPG4 hIgE (left) and α-CSPG4 rIgE (right) to FcϵRI-expressing RBL-SX38 (left) and RBL-2H3 cells (right) detected by flow cytometry. Representative results of one of four (RBL-SX38) and one (RBL-2H3) independent experiments. E, Binding profiles of commercial polyclonal anti-rat CSPG4 (AB5320), α-CSPG4 rIgE and α-CSPG4 hIgE to CSPG4-expressing rat C6 cells. F, Competition between increasing concentrations of rabbit AB5320 antibody (or MOv18 hIgE as control) and α-CSPG4 rIgE (detected with a secondary anti-rat IgE antibody) binding to C6 cells. G, RBL-2H3 cells degranulation in the absence of stimuli (HBSS), in the presence of α-DNP rIgE or α-CSPG4 rIgE alone, or upon crosslinking with α-IgE secondary antibody. Cells treated with Triton-X100 were set as 100%. Bars indicate mean ± SD from 3 independent experiments. **p < .01 by one-way ANOVA. H and I, RBL-2H3 cells-mediated ADCC against CFSE-labeled A2058 cells pre-coated with α-CSPG4 rIgE or α-DNP rIgE. H, Representative dot plot obtained in the presence of α-DNP rIgE (left panel) or α-CSPG4 rIgE (right panel). The percentage of dead cells was defined as DAPI+ A2058 cells (red box)*100/total A2058 cells (purple box). I, ADCC data obtained from 3 independent experiments. Bars indicate mean + SD; *p < .05 by one-way ANOVA. J, A2058 cells proliferation after incubation with α-CSPG4 rIgE or α-DNP rIgE (4 days). Incubation with no antibody was set as 100%. Data are the mean of 3 independent replicates.

### Generation and functional characterization of α-CSPG4 rIgE

We successfully generated α-CSPG4 rIgE, a rat IgE containing the variable regions of the light and heavy chains derived from the murine anti-CSPG4 clone 225.28 (Supplemental Figure 1).^31^ SDS-PAGE analysis of α-CSPG4 rIgE confirmed the predicted molecular size in both non-reducing and reducing conditions (Figure 2A). Sodium dodecyl sulfate-polyacrylamide gel electrophoresis (SDS-PAGE) and high performance liquid chromatography-size exclusion chromatography (HPLC-SEC) profiles of α-CSPG4 rIgE were comparable to those of the previously-described rat antibody MOv18 rIgE,^15^ which recognizes the human FRα (Figure 2A, B). Flow cytometric analysis showed that α-CSPG4 rIgE was able to bind the human CSPG4-expressing melanoma cell line A2058 in a dose-dependent manner and similarly to its human counterpart, α-CSPG4 hIgE^31^ (Figure 2C), with 50% of the maximal mean fluorescence intensity reached by the two antibodies at similar concentration (1.57 µg/ml α-CSPG4 rIgE and 1.11 µg/ml α-CSPG4 hIgE). Comparable binding of α-CSPG4 rIgE to the rat FcϵRI-expressing basophilic RBL-2H3 cells and α-CSPG4 hIgE to the human FcϵRI-expressing RBL-SX38 cells was observed (Figure 2D). The binding of the α-CSPG4 rIgE to the rat CSPG4-expressing C6 glial cells^32^ was comparable to that exhibited by a commercial polyclonal anti-CSPG4 antibody reactive for rat CSPG4 (Figure 2E) and the α-CSPG4 hIgE. Moreover, in a competition assay, α-CSPG4 rIgE binding to C6 cells was inhibited by the presence of increasing concentrations of the anti-rat CSPG4 antibody, confirming target antigen-binding specificity (Figure 2F). We concluded that the variable region derived from the 225.28 antibody clone was cross-reactive with the rat orthologue of CSPG4.

To further characterize the functional properties of α-CSPG4 rIgE, we tested its ability to activate rat immune effector cells. Crosslinking α-CSPG4 rIgE bound to rat FcϵRI-expressing basophilic RBL-2H3 cells led to significant induction of degranulation (Figure 2G), equivalent to that observed using a commercial rat anti-DNP IgE. No degranulation was observed by either antibody in the absence of the crosslinking agent. Moreover, α-CSPG4 rIgE triggered RBL-2H3-mediated antibody-dependent cell-meditated cytotoxicity (ADCC) against A2058 cells (Figure 2H, I). No anti-tumor effect was observed in A2058 cells treated with up to 250 µg/ml α-CSPG4 rIgE (Figure 2J). These findings indicated that the Fc region of α-CSPG4 rIgE was structurally functional, with ability to induce immune effector functions.

### Immediate reactions to α-CSPG4 rIgE administration

We next investigated the safety profile of α-CSPG4 rIgE in an immunocompetent rat model. Rats were injected via the tail vein with a single dose of 10 mg/kg α-CSPG4 rIgE, MOv18 rIgE or phosphate-buffered saline (PBS). Clinical signs were monitored for 1 hour after injection and observations summarized based on a previously reported scoring system (Supplementary Table 2).^15^ None of the animals treated with PBS presented abnormal or adverse effects, and injection of MOv18 rIgE induced mild responses in agreement with previous findings (Table 1).^15^ Following α-CSPG4 rIgE injection, transient mild or moderate adverse reactions were observed within 5 minutes, namely labored respiration, prostration, piloerection, reduced responsiveness and no peer interactions, but no severe reactions were observed. Symptoms resolved within 30 minutes from injections. Within the α-CSPG4 rIgE-treated group, we did not observe differences in the reactions of animals whether they were previously injected with untransfected CC531 or transfected CC531-hCSPG4 cells (Table 1, Supplemental Figure 3), indicating that the reactions observed were not due to the antibody encountering ectopically-expressed human CSPG4. A notable symptom observed in the α-CSPG4 rIgE-treated animals was that they started drinking eagerly ~ 20 to 30 minutes after injection.^33^

**Figure 3.**
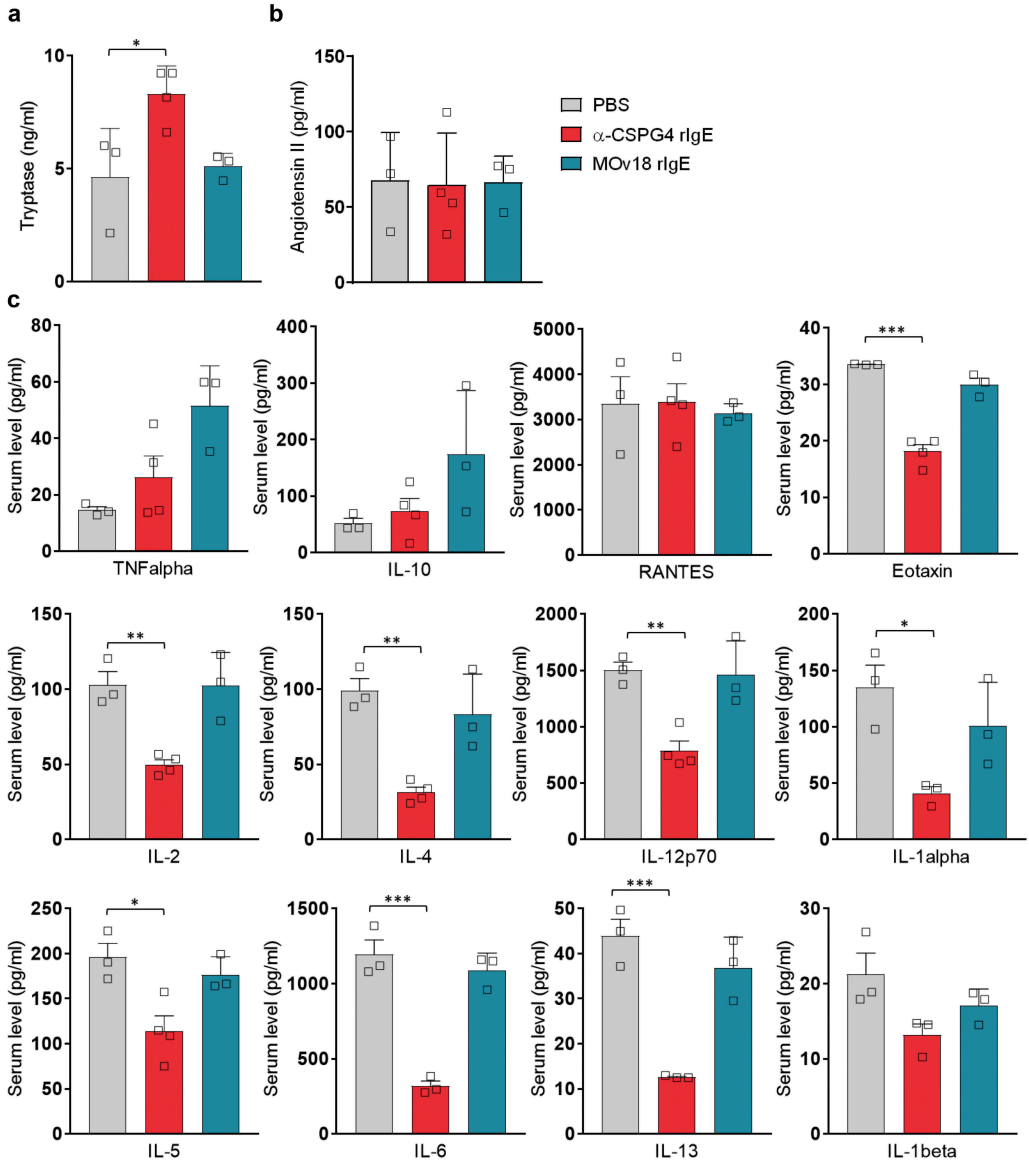
Molecular analysis of immediate effects of in vivo α-CSPG4 rIgE administration. A-B, Levels of tryptase (ng/ml) and angiotensin II (pg/ml) were measured via ELISA in the sera of WAG rats 30 minutes after intravenous administration of 10mg/ml α-CSPG4 rIgE (n = 4), 10mg/ml MOv18 rIgE (n = 3) or equivalent volume of PBS (n = 3). Bars represent average values ± SD; *p < .05 calculated using One-way ANOVA. C, Levels of selected cytokines (pg/ml) were measured using bead-based multiplex assay in the sera of WAG rats 30 minutes after intravenous administration of α-CSPG4 rIgE (n = 4), MOv18 rIgE (n = 3) or PBS (n = 3). Bars represent average values ± SD; *p < .05, **p < .01, ***p < .005 calculated using one-way ANOVA.

**Table 1.** Clinical signs observed following α-CSPG4 rIgE, MOv18 rIgE or PBS administration.

	PBS^a^ (n = 4)	MOv18 rIgE^a^ (n = 4)	α-CSPG4 rIgE^a^ (n = 4)	α-CSPG4 rIgE^b^ (n = 3)
Drinking	Normal	Normal	Abnormal behaviour	Abnormal behaviour
Piloerection	Normal	Mild	Moderate	Moderate
Responsiveness	Normal	Normal-Mild	Moderate	Moderate
Peer interaction	Normal	Normal	Moderate	Moderate
Hunching	Normal	Normal	Mild	Mild
Vocalisation	Normal	Normal	Normal	Normal
Oculo-nasal discharge	Normal	Normal	Normal	Normal
Respiration	Normal	Normal	Moderate	Moderate
Tremors	Normal	Normal	Normal	Normal
Convulsions	Normal	Normal	Normal	Normal
Prostration	Normal	Normal	Moderate	Moderate
Self-mutilation	Normal	Normal	Normal	Normal

^a^CC531-hCSPG4 cells injected via tail vein 24 hours before antibody administration.

^b^CC531 cells injected via tail vein 24 hours before antibody administration.

To gain insight into the molecular mechanisms linked with the adverse symptoms observed, we analyzed hypersensitivity biomarkers 30 minutes after antibody injection.^33^ Animals injected with MOv18 rIgE and PBS showed similar serum levels of the serine esterase tryptase (PBS: 4.6 ng/ml ± 1.2; MOv18 rIgE: 5.1 ng/ml ± 0.3; *p* = .97), whereas we observed higher levels of serum tryptase in animals treated with α-CSPG4 rIgE (8.3 ng/ml ± 1.2; *p* = .036) (Figure 3A). Levels of serum angiotensin II, a vasoconstrictor thought to be endogenously released following anaphylactic reaction to counteract the vasodilation,^33^ did not differ between treatments 30 minutes after injection (PBS: 67.4 pg/ml ± 18.4 MOv18 rIgE: 66.3 pg/ml ± 9.9; α-CSPG4 rIgE: 64.3 pg/ml ± 17.3) (Figure 3B). No significant differences were observed in serum levels of interleukin (IL)-10 and tumor necrosis factor (TNF) of α-CSPG4 rIgE-treated animals when compared to PBS (Figure 3C). Similarly, the levels of RANTES did not differ between the different groups. However, the serum levels of eotaxin, IL-2, IL-4, IL-12p70, IL-1α, IL-5, IL-6 and IL-13 decreased in the sera of animals treated with α-CSPG4 rIgE compared to PBS (Figure 3C). In particular, the Th2 type cytokines IL-6, IL-13, IL-4 and IL-1α showed ~70% decrease. Consistent with previous reports,^15,19^ we observed a partial increase in the levels of IL-10 and TNF following treatment with MOv18 rIgE (Figure 3C).

### Long-term safety profile of α-CSPG4 rIgE

**Figure 4.**
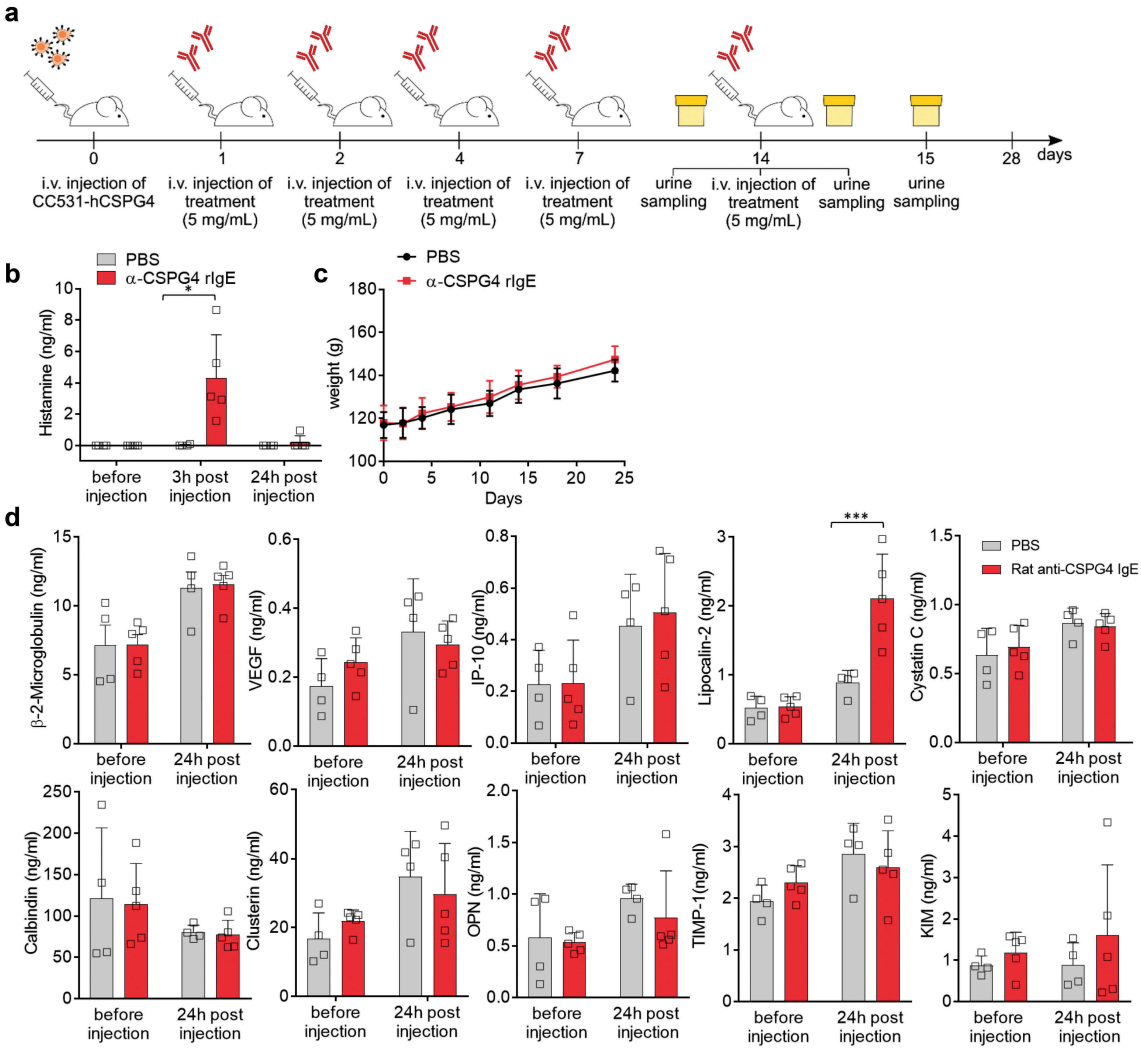
Effects of repeated α-CSPG4 rIgE administrations. A, Schematic representation of scheduling of α-CSPG4 rIgE administrations and urine sampling. Urine was collected immediately before, 3 hours and 24 hours after the last administration of 5mg/ml α-CSPG4 rIgE (n = 5) or PBS (n = 4). B, Urine levels of histamine (ng/ml) were measured in the urine at the three time points indicated in A. Bars represent average values ± SD; *p < .05 was calculated using two-way ANOVA. C, Body weight of rats during α-CSPG4 rIgE treatment indicated in A. Bars represent average values ± SD. D, Urine levels of selected markers for kidney function were measured using bead-based multiplex assay at the time points indicated. Bars represent average values ± SD; ***p < .005 calculated using two-way ANOVA.

We next sought to investigate the effects of repeated exposure to α-CSPG4 rIgE by injecting 5 mg/kg of α-CSPG4 rIgE on days 1, 2, 4, 7 and 14. Urine samples were collected immediately before, 3 hours and 24 hours after the last α-CSPG4 rIgE administration (Figure 4A).

After α-CSPG4 rIgE injection on day 14, the animals showed immediate adverse reactions as described above accompanied by the release of histamine, which was detected in the urine of α-CSPG4 rIgE-treated animals (4.31 ± 2.77 ng/ml) and dropped below the limit of detection (0.098 ng/ml) after 24 hours (Figure 4B). Repeated α-CSPG4 rIgE administrations did not affect animal body weight, which was comparable between treatments (Figure 4C). No differences in the urine levels of kidney function markers, namely β-2-microglobulin, calbindin, clusterin, interferon-inducible protein 10 (IP-10), osteopontin, kidney injury molecule-1, cystatin C, TIMP-1 or vascular endothelial growth factor were detectable between treatment groups before and 24 hours after the last injection (Figure 4D), indicating normal function. Notably, urine levels of lipocalin-2 (LCN2) were increased 24 hours after α-CSPG4 rIgE administration (Figure 4D).^34^

**Figure 5.**
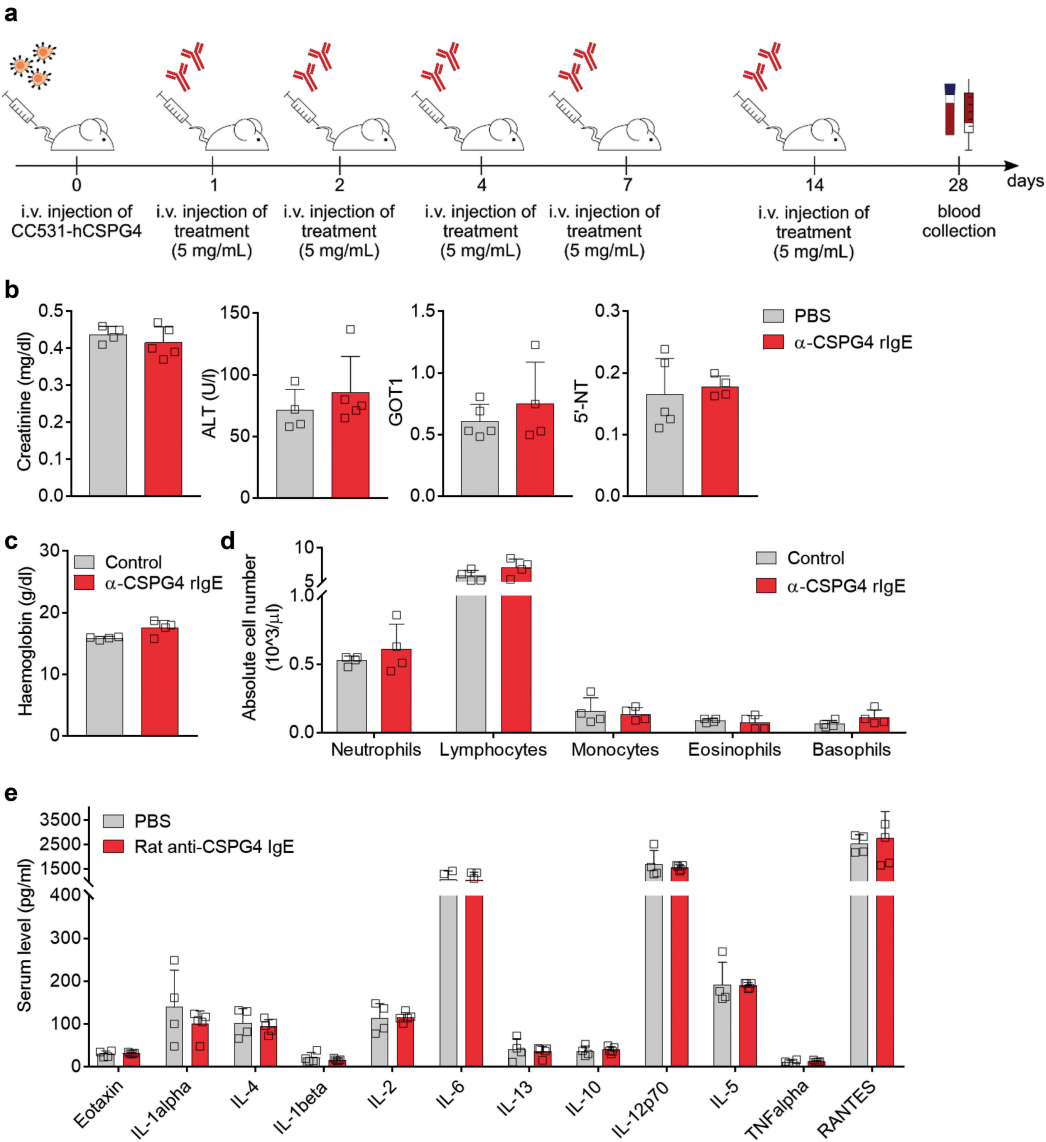
Long-term α-CSPG4 rIgE does not affect blood hematological or biochemical parameters. A, Blood of WAG rats treated with repeated α-CSPG4 rIgE (n = 5) or PBS (n = 4) administrations was collected before necropsy on experimental day 28. B, Serum levels of creatinine, alanine aminotransferase (ALT), Glutamic-Oxaloacetic Transaminase 1 (GOT1), 5ʹ-nucleotidase (5ʹ-NT). Bars represent average ± SD. Each symbol represents one rat. C, Hemoglobin concentration in the blood of WAG rats treated with α-CSPG4 rIgE or untreated (control). Bars represent average + SD. Each symbol represents one rat. D, Absolute blood cells numbers detected in α-CSPG4 rIgE or untreated animals. Bars represent average values ± SD. Each symbol represents one rat. E, Serum levels of selected cytokines (pg/ml) in α-CSPG4 rIgE or PBS treated animals. Bars represent average values ± SD.

At experimental day 28, animals were sacrificed, and blood samples were collected (Figure 5A). We did not observe changes in the serum levels of the liver damage-associated markers creatinine, alanine transaminase, 5ʹ-nucleotidase or glutamic-oxaloacetic transaminase (Figure 5B). Similarly, blood hemoglobin concentration and blood cell counts (Figure 5C, D) were comparable between groups. Levels of serum cytokines, found altered immediately after α-CSPG4 rIgE treatment, did not differ between treatment groups at day 28 (Figure 5E). These data indicate that repeated α-CSPG4 rIgE administrations did not induce signs of long-term toxicity.

### Discussion

The ongoing Phase 1 clinical study (NCT02546921, www.clinicaltrials.gov) of MOv18, a first-in-class therapeutic IgE antibody for ovarian cancer therapy, represents a substantial advance for the field, and it underlines the importance of further exploration of the safety profile and clinical applicability of IgE therapeutics against different tumor targets.

Here, we investigated the *in vivo* safety of a surrogate antibody of the IgE class in immunocompetent rats, chosen because of the similarities in the structure and immune distribution of human and rat FcϵRI, as previously described.^15,24^ Moreover, the antibody tested here recognizes the rat orthologue of its human target, CSPG4, making this model particularly relevant, as antigen cross-reactivity has not until now been reported in the context of IgE-based treatments.

CSPG4 is a promising target antigen highly expressed in multiple tumor types, including melanoma, glioblastoma, breast carcinomas, osteosarcoma and hematologic cancers.^25,35^ Originally believed to have a very limited distribution in normal adult tissues,^36^ CSPG4 expression has since been observed in a wider range of organs, but preferentially associated with progenitor cell phenotypes.^37^ Accordingly, we detected low to moderate expression of CSPG4 in some human anatomical locations including the CNS, spleen, bone marrow and endocrine organs like the thyroid gland and adenohypophysis. The distribution of the rat orthologue of CSPG4 in normal rat tissues was comparable to that observed in humans, as detected by screening different anatomical locations using tissue microarrays.

At present, despite the large amount of preclinical data on the anti-tumor efficacy of CSPG4-targeting therapies, few clinical studies have been reported. Early-phase clinical trials conducted in patients with malignant melanoma showed that immunization with the CSPG4 mouse anti-idiotypic antibody MK2-23 induced the production of anti-CSPG4 antibodies and correlated with survival prolongation without reporting target-related toxicity.^38^ Similarly, intravenous injection of the ^213^Bi-cDTPA-9.2.27 immunotherapeutic agent, based on the mouse anti-CSPG4 9.2.27 mAb and the ^213^Bi α-particle-emitting radioisotope, did not cause adverse events when administered to metastatic melanoma patients up to a dose of 925 MBq in a Phase 1 clinical trial.^39^ In light of the possibility of on-target/off-tumor toxicities due to the expression of CSPG4 in normal tissues, and based on known toxicity effects of systemically-administered CAR-T cell therapies, intracranial (intratumor) delivery was adopted instead of intravenous administration in a recent study on CAR-T cells based on the anti-CSPG4 mAb clone 763.74 in a xenograft murine model of glioblastoma.^40^

Here, we attempted to recreate a model for the study of on-target/off-tumor toxicity effects, which raise particular concern in systemically administered therapeutic agents.^41^ Using stable transfection, we produced a functionally-active surrogate rat IgE antibody, α-CSPG4 rIgE, based on the constant regions of rat IgE and the variable regions of the anti-CSPG4 murine antibody 225.28.^42,43^ The α-CSPG4 rIgE was able to induce mast cell degranulation upon crosslinking and to mediate tumor cell killing upon binding to rat FcϵRI-expressing effector cells, attributes previously shown with other IgE antibodies targeting cancer antigens.^10,44,45^

Intravenous injection of 5–10 mg/kg α-CSPG4 rIgE into Wistar Albino Glaxo (WAG) rats, with dosage selected based on previous studies of safety and anti-tumor efficacy of MOv18 IgE in a similar rat model,^15^ induced temporary mild to moderate adverse effects, such as labored respiration, prostration, piloerection and reduced responsiveness, which resolved within 30 minutes of injection. The reactions were observed in α-CSPG4 rIgE-treated animals regardless of the ectopic expression of the human target in previously-inoculated cell line CC531. As we demonstrated that α-CSPG4 rIgE recognizes the rat orthologue of CSPG4, we hypothesized that the interaction of the antibody with the rat tissues could be responsible for the transient reactions observed. Moreover, our control antibody MOv18 rIgE, which is thought not to recognize the rat homologue of its human target antigen FRα, did not show the same pattern of clinical signs. On a molecular level, we detected slightly higher levels of serum tryptase^46^ in α-CSPG4 rIgE-treated animals compared with PBS and MOv18 rIgE controls 30 minutes after the treatment and the presence of histamine in the urine 3 hours after antibody injection, which then dropped below the detection limit after 24 hours. These may indicate a temporary IgE-mediated activation of basophils and/or mast cells that self-resolved completely. This process was not accompanied by any increases in the levels of serum angiotensin II or cytokines, such as TNF, IL-2, IL-4, IL-5, IL-6, IL-10 and IL-13 among others, normally reported as a concomitant event during anaphylactic reactions.^33,47,48^ Conversely, most of the cytokines tested here were significantly downregulated 30 minutes after the injection of α-CSPG4 rIgE. This was the case for IL-2, IL-4, IL-5, IL-6, IL-13, IL-1α, IL-12(p70) and eotaxin (CCL-11). As blood withdrawal was performed after sacrificing the animals, our serum cytokine analysis was limited by the lack of multiple time-points. Further studies will be needed to dissect the molecular interplay and the dynamics of the immune mediators involved. Interestingly, we found significantly elevated LCN2 levels in the urine of α-CSPG4 rIgE-treated rats 24 hours after antibody administration. Besides its role as a biomarker of acute kidney injury,^49^ LCN2 was identified as mediator of the innate immune response^50^ and a protective component during the development of airway hyperreactivity and inflammation in a murine model of allergic airway.^34^ Moreover, elevated serum levels of LCN2 were found to be associated with improvement in the quality of life of patients with chronic urticaria.^51^ In light of these studies, our data may indicate a specific role for LCN2 in the IgE-mediate immune response, possibly by acting as a negative feedback in the inflammatory cascade.

Long-term, repeated antibody treatment did not affect the general health of the animals; their weight, blood cell counts, and metabolic markers of kidney and liver functions were comparable to the control group. Similarly, when tested 7 to 14 days after antibody injection, the levels of the cytokine and biomarkers in serum and urine were not different compared to controls.

In summary, our study provides the safety profile of passive IgE immunotherapy in a rodent species that features comparable expression of the target antigen and antibody cross-reactivity between the target human antigen and the orthologue of the host species. As administration of high doses (5–10 mg/kg) of α-CSPG4 rIgE induced only temporary mild to moderate adverse effects, our findings support the rationale for evaluating IgE-based compounds as immunotherapeutic agents. As the pharmacological translation from rodent to human is limited by the lack of knowledge on several aspects of rodent immunology, further insights into the safety of IgE-based immunotherapy will be provided by future studies on primates and reports from the current clinical study. Here, the comparable distribution of CSPG4 in human and rat normal tissues renders this system a useful platform for proof-of-concept studies on the safety and toxicology of different biological and immune therapies targeting CSPG4, including those based on the 225.28 clone, the efficacy of which has been extensively described against different solid tumors and in several formulations.^52–55^ By remodeling the tumor microenvironment and harnessing anti-tumor surveillance by immune effector cells such as macrophages, IgE antibodies may overcome tumor-induced immunomodulating forces that restrict the effectiveness of IgGs in tumor-targeted antibody therapy.^15,19,20,56^ On the other hand, the faster clearance of IgEs compared to IgGs could represent a challenge in terms of tumor penetration and pharmacokinetic properties and should be further investigated.^6^ Whether the higher affinity of IgE to its Fcϵ receptors compared to that of IgGs for cognate Fcγ receptors can compensate for a lower concentration of antibody at the tumor site still need to be determined. Future studies focused on IgE biodistribution will help to a better understand the potential of this class and its applicability in different therapeutic indications.

## Materials and methods

### Cell culture

The human Expi293F^TM^ cell line (ThermoFisher Scientific) was grown in Expi293F expression medium (Gibco) under shaking condition (125 rpm) at 8% CO₂ in a 37ᵒC humidified incubator. All other cell lines used were maintained in 37ᵒC humidified 5% CO₂ incubator. Human melanoma cell line A2058 and rat glial C6 cells (ATCC) were grown in DMEM (Gibco) supplemented with 10% and 15% fetal bovine serum (FBS) (Gibco) respectively. Rat FcϵRI-expressing basophilic RBL-2H3 cells (ATCC) were grown in MEM (Gibco) containing 15% FBS. Human FcϵRI-expressing basophilic rat RBL-SX38 cells (ATCC) were grown in RPMI1640 (Gibco) medium containing 10% FBS. The WAG rat-derived colon adenocarcinoma cell line CC531 cells (CLS Cell Lines Service GmbH) were grown in RPMI1640 (Gibco) containing 10% FBS.

CC531 was transfected with a pDEST26 vector encoding for the full-length human CSPG4 (Source Bioscience) using Lipofectamine 2000 according to manufacturer’s instructions. Cells were maintained under selection with 200 μg/ml G418 antibiotic (GE Healthcare) for 4 weeks, then stained with allophycocyanin (APC)-conjugated anti-CSPG4 (clone: REA1041, Miltenyi Biotec) and APC+ cells (CC531-hCSPG4) were sorted using a BD FACSAria cell sorter (BD Biosciences). The WAG rat-derived colon adenocarcinoma cell line CC531 was transfected with a pDEST26 vector encoding for the full-length human CSPG4 (Source Bioscience) using Lipofectamine 2000 according to manufacturer’s instructions. Cells were maintained under selection with 200 μg/ml G418 antibiotic (GE Healthcare) for 4 weeks, then stained with APC-conjugated anti-CSPG4 (clone: REA1041, Miltenyi Biotec) and APC+ cells (CC531-hCSPG4) were sorted using a BD FACSAria cell sorter (BD Biosciences).

### Immunohistochemistry analysis of CSPG4 expression

The multiple organ normal tissue microarray FDA999g was purchased from US Biomax. Tissues were de-waxed with Xylene, followed by rehydration with ethanol. Heat-induced antigen retrieval was performed in a 95°C water bath, using citric acid. Subsequently, sections were stained following standard immunohistochemistry (IHC) procedures using the commercial mouse monoclonal anti-CSPG4 antibody (clone: LHM-2, Abcam). IHC detection of orthologue rat CSPG4 on the paraffin-embedded rat normal tissue array (RAT901A) was performed by US Biomax using the commercial rabbit polyclonal anti-NG2 antibody (AB129051, Abcam).

### Gene expression analysis

CSPG4 gene expression data in different species were obtained from the databases indicated through interrogation of EBI search.^57^ When more than one dataset was available, data were presented as mean ± SD using GraphPad Prism.

### Cloning and production of recombinant antibodies

For generation of an anti-CSPG4 rat/mouse chimeric IgE (α-CSPG4 rIgE), codon-optimized gBlock gene fragments (Integrated DNA Technologies), one encoding for the anti-CSPG4 antibody (clone 225.28) heavy chain variable region fused to the rat epsilon heavy chain constant domains, and the other encoding for 225.28 light chain variable region fused to the rat kappa light chain constant domains, were cloned into two separate UCOE Mu-H plasmids (Merck Millipore) using polymerase incomplete primer extension as previously described (Supplemental Figure 1; Supplemental Table 1).^31,42,58^ Expi293F cells were co-transfected with the two vectors using Lipofectamine 2000 (Invitrogen) according to manufacturer’s instructions. Culture supernatant was tested for α-CSPG4 rIgE secretion via A2058 cells staining as described in the Supplemental Methods. For antibody production, cells were seeded at 2x10^7^/ml and cultured for 24h. The antibody was purified from supernatant with HiTrap Protein L affinity columns (GE Healthcare) and dialyzed against PBS using Tube‐O‐DIALYZER (G-Biosciences). The anti-CSPG4 human/mouse chimeric IgE antibody (α-CSPG4 hIgE), also based on the mouse antibody clone 225.28, and the anti-FRα rat MOv18 IgE (MOv18 rIgE) were produced as described.^15,31^

### Analysis of antibody purity

Antibody purity was verified by SDS-PAGE and SEC. For SDS-PAGE, purified antibodies (5μg) were mixed with Laemmli sample buffer (Bio-Rad Laboratories) with or without the reducing agent dithiothreitol (DTT, final concentration 100 μM) (Bio-Rad Laboratories) and heated at 95ᵒC for 5min. Samples and protein ladder (PageRuler Plus 10-250kDa, ThermoFisher Scientific) were then run on a 4–15% Mini-PROTEAN TGX gel (Bio-Rad Laboratories) and stained with Coomassie dye (Expedeon). For SEC, antibodies were analyzed using a Superdex 200 10/300 GL column (GE Healthcare) using a Gilson HPLC system.

### Flow cytometry

To test in-house produced antibody binding to CSPG4, cells were detached using 5 mM ethylenediaminetetraacetic acid (EDTA) in PBS, resuspended at 1 × 10^6^ cells/ml and incubated on ice for 30 min with the indicated concentration (10 µg/ml, if not specified) of primary antibodies in fluorescence-activated cell sorting (FACS) buffer (2% FBS in PBS). Cells were then washed and incubated with fluorescently-labeled secondary antibody in FACS buffer for further 30 min, washed twice and analyzed using a BD FACSCanto II flow cytometer (BD Biosciences). For competition binding assay, C6 cells were incubated with increasing concentrations of unlabeled competitor rabbit polyclonal anti-rat CSPG4 antibody (AB5320, Abcam) or MOv18 hIgE as isotype control together with 5 µg/ml of the in-house produced rat anti-CSPG antibody, which was detected with a fluorescein isothiocyanate (FITC)-conjugated anti-rat IgE (MA516812, Invitrogen). The commercial APC-conjugated anti-human CSPG4 (clone: REA1041, Miltenyi Biotec) was used as a positive control to detect CSPG4 in human cell lines. The FITC-conjugated anti-human IgE (FI-3040, Vector Laboratories) was used as a secondary antibody to detect in-house produced human IgEs.

### Cell proliferation assay

A2058 cells were seeded in 96-well plates (1x10^3^/well) and incubated with varying concentrations of antibody or PBS in growing media to a final volume of 100 μl. After 4 days, media was replaced with 100 μl of fresh media containing CellTiter96 AQueousOne Solution Reagent (G3582, Promega) according to manufacturer instructions. Cells were then incubated for 2 hours at 37ᵒC in the dark in a humidified 5% CO_2_ incubator. Absorbance at 490 nm and background at 690 nm was read using a FluoStar Omega microplate reader (BMG Labtech). The relative cell proliferation (%) was calculated as the absorbance of antibody-treated cells/absorbance of PBS-treated cells.

### Bead-based multiplex and ELISA assays

Levels of selected cytokines in the rat serum were measured by bead-based Multiplex assay kit (RECYTMAG-65K, Millipore) according to the manufacturer’s instructions using a FlexMap 3D (Luminex). Serum levels of angiotensin II and tryptase were measured via ELISA kits (CSB-E04494r, CSB-E13627r, Cusabio Technology). Biomarkers of kidney-related toxicity were measured in the rat urine by bead-based Multiplex assay kit (RKTX1MAG-37K and RKTX2MAG-37K, Millipore) according to the manufacturer’s instructions. ELISA kit (ab213975, Abcam) was used to determine histamine levels in the rat urine.

### Antibody-induced cell degranulation

The IgE-mediated RBL-2H3 cell degranulation assay was performed according to a previously described method^59^ with modifications. RBL-2H3 cells (1x10^4^/well) were incubated with 1 μg/ml of in-house produced α-CSPG4 rIgE, a commercial rat monoclonal anti-DNP IgE (MA5-16776, Invitrogen) or left untreated for 1hr at 37ᵒC. Cells were then washed with HBSS (Gibco) and incubated with either HBSS, 0.1% Triton-X100 in HBSS or 1 μg/ml of polyclonal goat anti-rat IgE (PA1-29379, ThermoFisher Scientific) as crosslinker for 2 hr. An aliquot of supernatant (50 μl) was then diluted 1:1 in HBSS and incubated with 50 μl of fluorogenic substrate (final concentration: 0.5 mM 4-methylumbelliferyl N-acetyl-β-D-glucosaminide, 0.05% DMSO, 0.05% TitonX-100, 100 mM citrate buffer pH4.5) for 2 hr at 37ᵒC in a Nunc-Immuno™ black 96-well plate. The reaction was quenched with 1:1 0.5 M Tris-HCl pH 8.2 and fluorescence detected using a FluoStar Omega microplate reader (BMG Labtech) with excitation and emission wavelengths of 350 nm and 450 nm.

### Antibody-dependent cell-mediated cytotoxicity

A2058 cells (target) were stained in PBS containing 5 μM CellTrace™ CFSE (ThermoFisher Scientific). After 24 h, cells were detached with 5 mM EDTA in PBS and incubated with the desired antibody (10 μg/million cells) or an equivalent volume of PBS at 37ᵒC for 30min. A2058 cells were then washed, resuspended at 5x10^5^/ml in 100 μl of MEM containing 2% FBS and mixed with previously trypsinized RBL-2H3 cells (effector) at 1:1 effector:target ratio. After 2 h incubation at 37ᵒC in 5% CO_2_, DAPI (ThermoFisher Scientific) was added to the samples and the fluorescence was acquired using a BD FACSCanto II flow cytometer (BD Biosciences). Cells were gated based on forward and side scatter, and quadrant gates were applied to determine the % DAPI+ A2058 cells (DAPI^+^ CFSE^+^ *100/total CFSE^+^).

### Immunocompetent WAG rat model

Female WAG/RijCrl rats 6–8 weeks old were purchased from Charles River. All procedures were performed in accordance with the Institutional Committees on Animal Welfare of the UK Home Office and under The Home Office Animals Scientific Procedures Act, 1986. Antibodies at the specified concentration or an equivalent volume of PBS were injected via the tail vein at the indicated time points. Where indicated, CC531 cells and CC531-hCSPG4 transfected cells (3–4 x 10^6^) were injected via the tail vein. Animals were monitored and symptoms were scored using scoring criteria reported in Supplemental Table 1. At the end of the experiments, animals were culled by asphyxiation with carbon dioxide and blood samples were collected. For serum isolation, blood samples in Microvette® tubes (Sarstedt) were spun at 10,000 g for 10 min and separated serum was stored at −80ᵒC. Blood collected into EDTA-containing tubes was used for cell counts. Measurement of serum hemoglobin, creatinine, alanine aminotransferase and blood cell counts were performed by Sequani Ltd. Urine was collected at the times indicated and stored at −80ᵒC.

### Statistical analysis

Data are presented as mean ± SD. For statistical comparison of one independent variable, one-way ANOVA with Tukey’s multiple comparisons test was calculated. For grouped data, two-way ANOVA was calculated and Sidak’s multiple comparisons test was applied. *P* values <.05 were considered statistically significant. Statistical analysis was performed using GraphPad Prism software (version 7.03).

## Data Availability

All data generated or analysed during this study are included in this published article and its Supplementary Material file.

## Supplementary Material

Availability of data and materials
