# Supplementary material for: In vivo safety profile of a CSPG4-directed IgE antibody in an immunocompetent rat model
Source: MAbs. 2019 Nov 26;12(1):1685349. doi: 10.1080/19420862.2019.1685349 (PMC6927758; doi:10.1080/19420862.2019.1685349)
Supplement: Supplemental Material [file kmab-12-01-1685349-s001.zip › Supplementary information/Supplemental_Material.docx]

**Supplemental Material**

**Supplemental Table I.** Primers used for PIPE cloning

| **Primer function** | **Primer name** | **Sequence (5’->3’)** |
| --- | --- | --- |
| **UCOE vector backbone linearization** | **U1 F** | **GTCGACCTGCAGGCATGCAAG** |
|  | **U1 R** | **CGTTGTCAGAAGTAAGTTGG** |
|  | **U2 F** | **CCAACTTACTTCTGACAACG** |
|  | **U2 R** | **AGCACTAACGAAGTAGAGGG** |
|  | **U3 F** | **CCCTCTACTTCGTTAGTGCT** |
|  | **U3 R** | **GGCCGGCCGTTAACTTAACTAAC** |
| **α-CSPG4 Kappa light chain gene fragment amplification** | **Rat CSPG4 LC F** | **AGTTAACGGCCGGCCATGTTG** |
|  | **Rat CSPG4 LC R** | **ATGCCTGCAGGTCGACCTAACAC** |
| **α-CSPG4 Epsilon heavy chain gene fragment amplification** | **Rat CSPG4 HE F** | **AGTTAACGGCCGGCCATGGACT** |
|  | **Rat CSPG4 HE R** | **ATGCCTGCAGGTCGACCTACATA** |

**Supplemental Table II.** Severity of clinical signs

|  | Severity of Clinical Sign | | |
| --- | --- | --- | --- |
| Clinical Sign Category | Mild | Moderate | Severe |
| Food and water consumption | 40 – 75% of normal for 72 hours | < 40% of normal for 72 hours | < 40% of normal for 7 days |
| Piloerection | Partial piloerection | Marked piloerection  (staring coat) | Marked piloerection (staring coat) with other signs of dehydration |
| Responsiveness | Subdued but responsive, normal provoked patterns of behaviour | Subdued with subdued behaviour patterns even when provoked | Unresponsive to extraneous activity and provocation |
| Peer interaction | Interacts with peers | Little peer interaction |  |
| Hunching | Hunched transiently especially after dosing | Hunched intermittently | Hunched persistently (‘frozen’) |
| Vocalisation | Transient vocalisation | Intermittent – vocalisation when provoked | ‘Distressed’ – vocalisation unprovoked |
| Oculo-nasal discharge | Transient | Persistent | Persistent and copious |
| Respiration | Normal respiration | Intermittent abnormal breathing pattern | Laboured respiration |
| Tremors | Transient | Intermittent | Persistent |
| Convulsions | None | Intermittent | Persistent |
| Prostration | None | Transient prostration  (< 1 hour) | Prolonged prostration  (> 1 hour) |
| Self-mutilation | None | None | Self-mutilation |

**
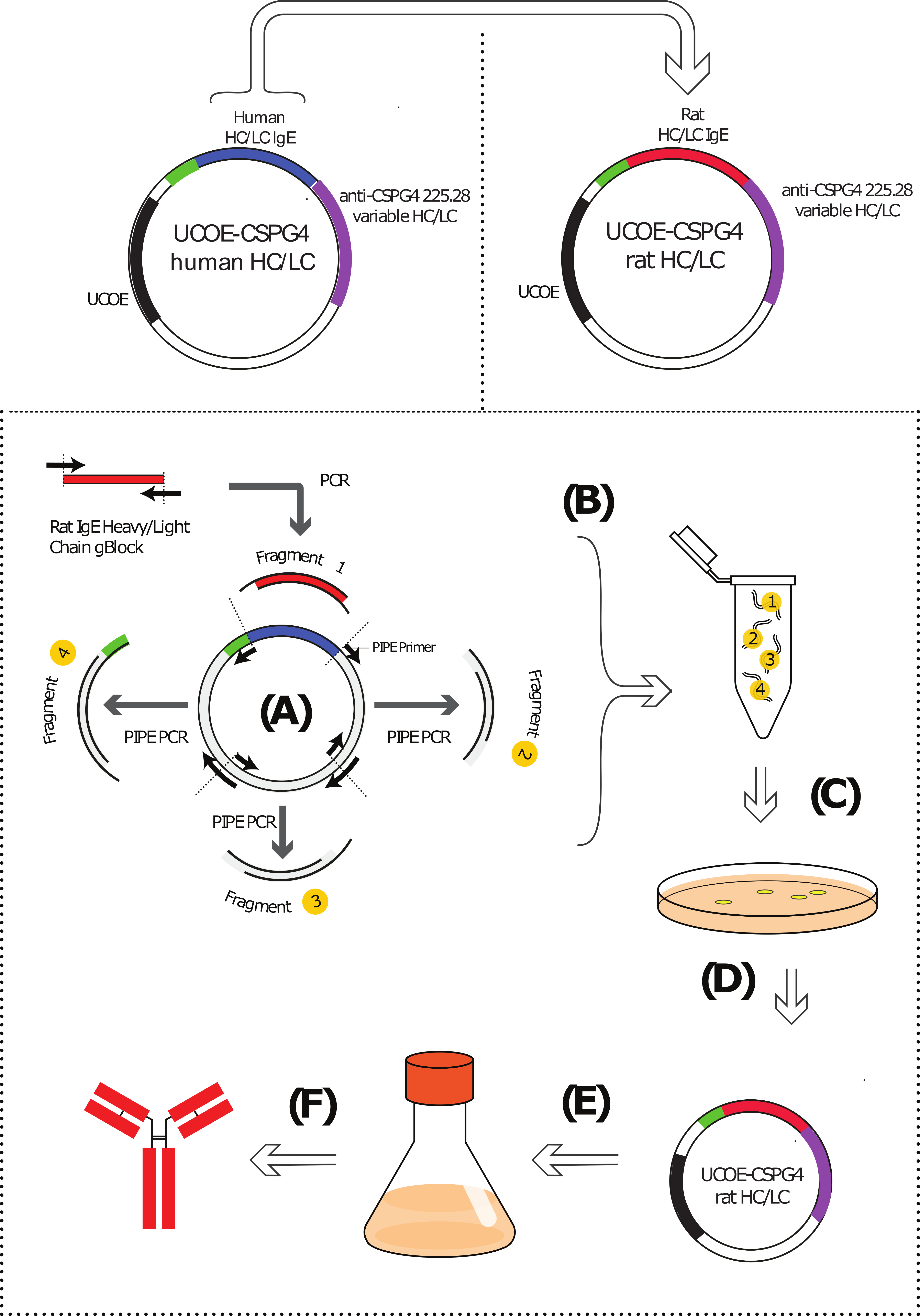
**

**Supplemental Figure 1.** Schematic representation of the cloning strategy used to generate α-CSPG4 rIgE. **A-D**, The PIPE method was used to clone the IgE rat constant heavy and light chains fused respectively to the heavy and the light chain of the murine antibody clone 225.28 into UCOE vectors. **E-F**, The resulting α-CSPG4 rIgE antibody was produced via stable transfection and purified through Protein L column.

**
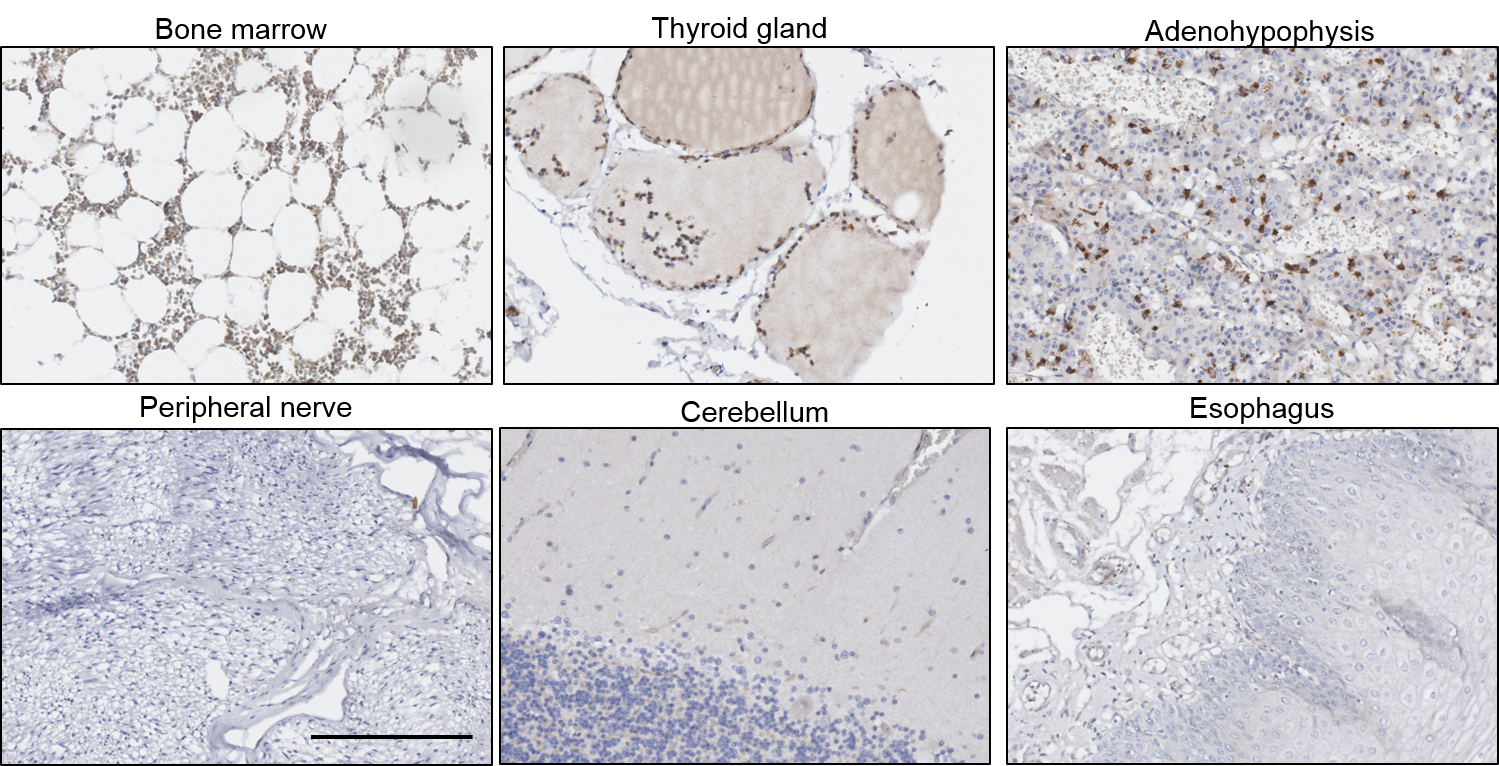
**

**Supplemental Figure 2.** CSPG4 expression in normal human tissues. CSPG4 expression was investigated by immunohistochemistry of tissue microarrays using a commercial anti-CSPG4 antibody and developed using DAB chromogen. Hematoxylin was used to counterstain. Scale bar represent 200μm.

**
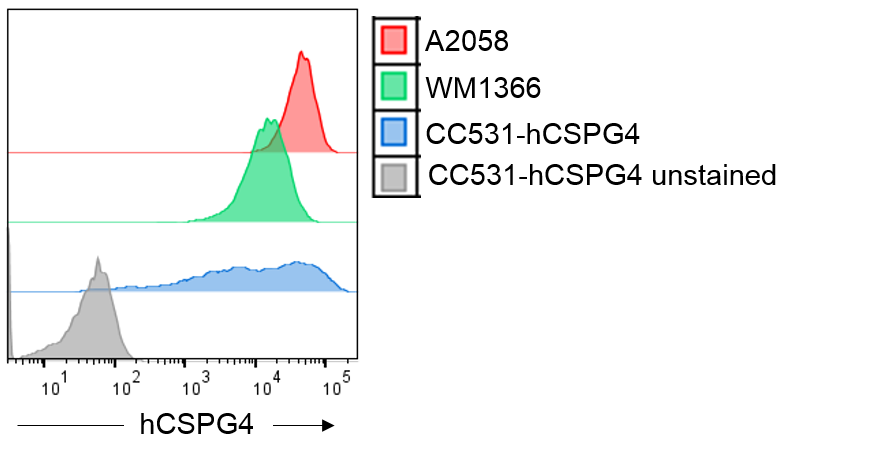
**

**Supplemental Figure 3.** Flow cytometry detection of CSPG4 in two melanoma cell lines (A2058, WM1366) and transfected CC531 cells (CC531-hCSPG4).
