# Supplementary figures and images for: In vivo safety profile of a CSPG4-directed IgE antibody in an immunocompetent rat model
Source: MAbs. 2019 Nov 26;12(1):1685349. doi: 10.1080/19420862.2019.1685349 (PMC6927758; doi:10.1080/19420862.2019.1685349)

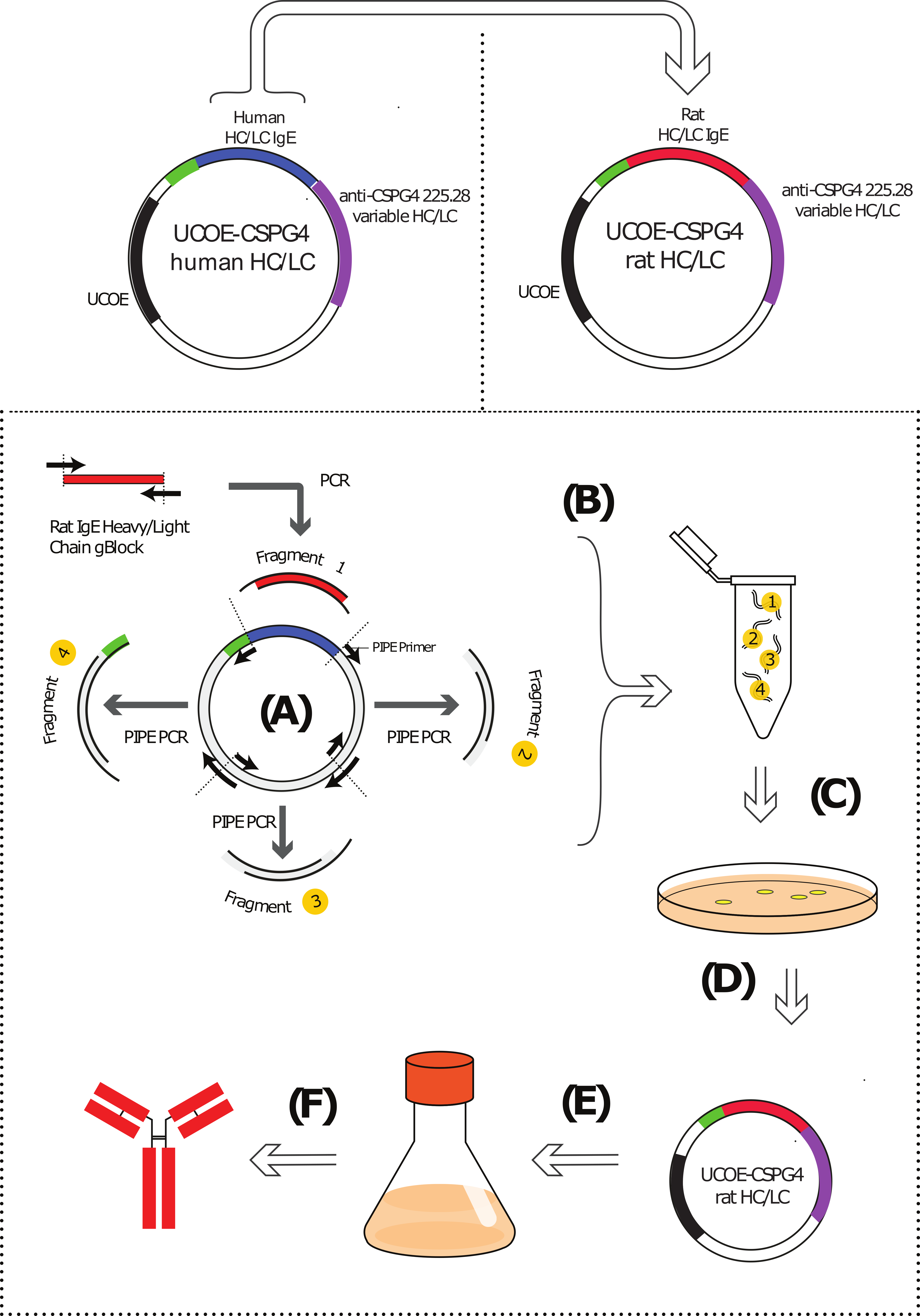

Supplement: Supplemental Material [file kmab-12-01-1685349-s001.zip › Supplementary information/Suppl_Fig1.png]

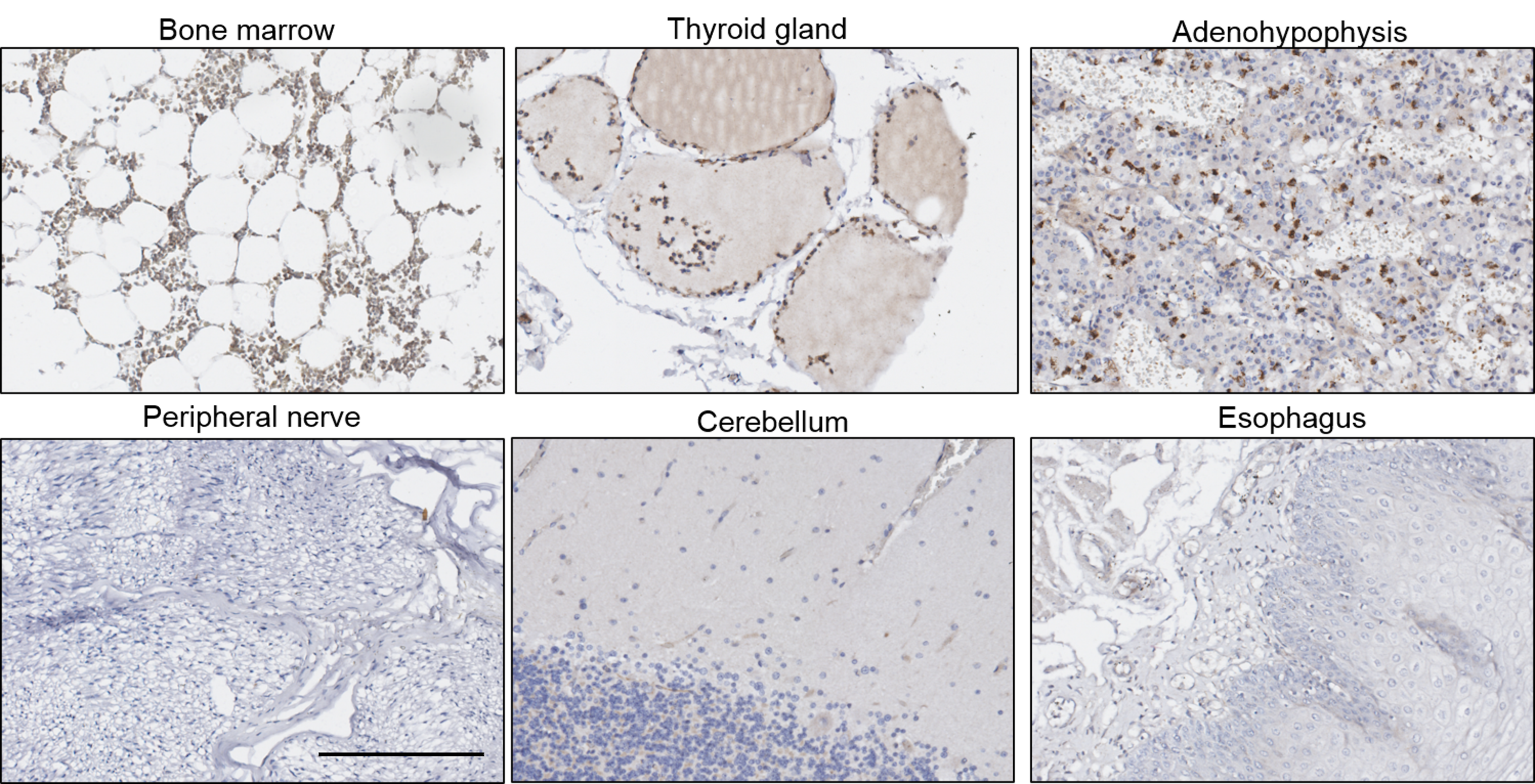

Supplement: Supplemental Material [file kmab-12-01-1685349-s001.zip › Supplementary information/Suppl_Fig2.tif]

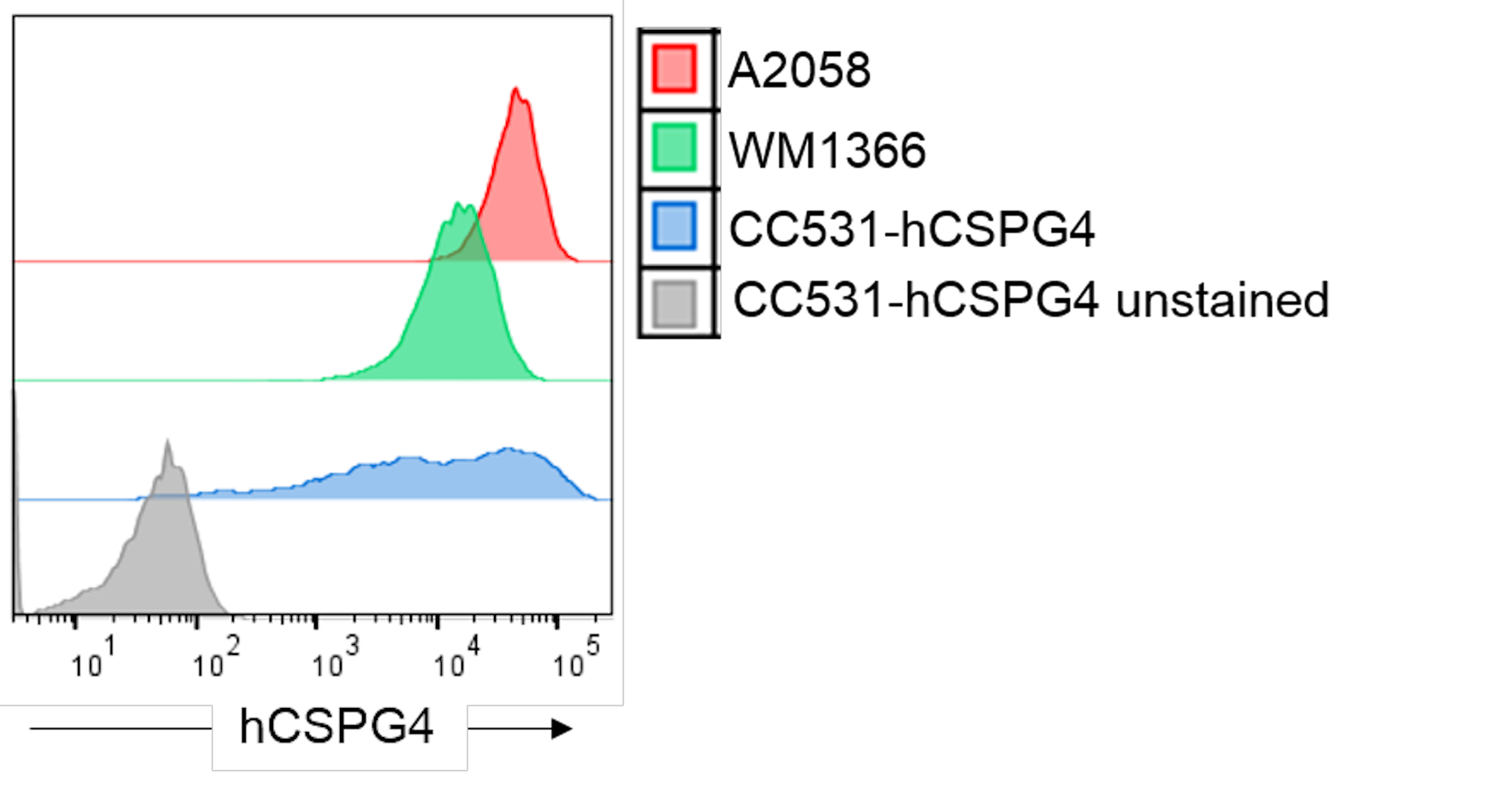

Supplement: Supplemental Material [file kmab-12-01-1685349-s001.zip › Supplementary information/Suppl_Fig3.tif]
